# Analyzing Malaria Disease Using Effective Deep Learning Approach

**DOI:** 10.3390/diagnostics10100744

**Published:** 2020-09-24

**Authors:** Krit Sriporn, Cheng-Fa Tsai, Chia-En Tsai, Paohsi Wang

**Affiliations:** 1 Department of Tropical Agriculture and International Cooperation, National Pingtung University of Science and Technology, Neipu, Pingtung 91201, Taiwan; krit22kmutt@gmail.com; 2 Department of Information Technology, Suratthani Rajabhat University, Suratthani 84100, Thailand; 3 Department of Management Information Systems, National Pingtung University of Science and Technology, Pingtung 91201, Taiwan; 4 Department of Biochemistry and Molecular Biology, National Cheng Kung University, Tainan 70101, Taiwan; plusntsai@gmail.com; 5 Department of Food and Beverage Management, Cheng Shiu University, Kaohsiung 83347, Taiwan; 0627@gcloud.csu.edu.tw

**Keywords:** activation function (Mish), image processing, image classification, malaria, convolutional neural network, optimization methods, deep learning

## Abstract

Medical tools used to bolster decision-making by medical specialists who offer malaria treatment include image processing equipment and a computer-aided diagnostic system. Malaria images can be employed to identify and detect malaria using these methods, in order to monitor the symptoms of malaria patients, although there may be atypical cases that need more time for an assessment. This research used 7000 images of Xception, Inception-V3, ResNet-50, NasNetMobile, VGG-16 and AlexNet models for verification and analysis. These are prevalent models that classify the image precision and use a rotational method to improve the performance of validation and the training dataset with convolutional neural network models. Xception, using the state of the art activation function (Mish) and optimizer (Nadam), improved the effectiveness, as found by the outcomes of the convolutional neural model evaluation of these models for classifying the malaria disease from thin blood smear images. In terms of the performance, recall, accuracy, precision, and F1 measure, a combined score of 99.28% was achieved. Consequently, 10% of all non-dataset training and testing images were evaluated utilizing this pattern. Notable aspects for the improvement of a computer-aided diagnostic to produce an optimum malaria detection approach have been found, supported by a 98.86% accuracy level.

## 1. Introduction

The World Health Organization (WHO), through an estimation of the demography in its World Malaria Report 2018, reported that there were 212 million patients and as many as 435,000 patient deaths worldwide from malaria. In tropical Africa, it is estimated that 3.1 billion US dollars are lost per year due to increased public health expenditures, adversely affecting tourism [1,2]. Malaria is a disease caused by the Plasmodium parasite that spreads throughout the human body through the bites of female anopheles, which can then spread to others from mosquitoes that bite malaria patients. However, it cannot spread from person to person. In addition to being transmitted from mother to fetus, patients may be infected with malaria through blood transfusions or through sharing syringes [3,4]. The symptoms of an infected person are similar to the flu and can also include other symptoms, such as a high fever, chills, septicemia, pneumonia, gastritis, enteritis, nausea, vomiting and death [5,6]. Malaria is often found in areas with hot, humid climates near natural water resources, representing the habitat of anopheles mosquitoes that carry contagious diseases [7,8].

**Figure 1:**
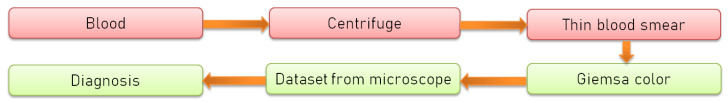
The dipstick method.

The method of malaria diagnosis consists of a centrifuge machine separating white blood cells and red blood cells so that only red blood cells can be used for analysis by employing a blood film. It is a standard laboratory method for diagnosing malaria and is known as a dipstick method for diagnosis, including thick and thin blood smears [9,10]. The method of detecting malaria by microscopy can report the results of the analysis in terms of both the amount and species of the infection to help diagnose malaria, but it is also useful in monitoring the treatment of patients. Malaria patients are diagnosed and treated without delay, and the doctor treats the patient by using antimalarial agents, such as Chloroquine, Doxycycline, Quinine Sulfate, Hydroxychloroquine and Mefloquine [11]. Thick and thin blood smears are a detected feature of red blood cells (RBCs) shown in blood films, revealing features such as the color, size, texture, morphology and position of the parasite from the malaria patient. They represent the most popular method for the diagnosis of malaria for all clinics, hospitals and medical laboratories because they represent an inexpensive method for the diagnosis of an endemic disease such as malaria [12,13]. Figure 1 presents the dipstick method.

The methods employed conduct deep investigations of blood smears by using a microscope, which provides images of patient‘s blood to the doctor or medical laboratory technologist for finding parasites in RBCs. Deep learning is a subset of biologically inspired machine learning methods that were designed to imitate the function of information processing and decision making in the human brain. Functions of the human brain are much wider than current deep learning capabilities and include organization, awareness, personality, etc [14]. Nowadays, there are many different research techniques that use deep learning for many of the most widely-used computer vision and pattern recognition and commercial applications. The convolutional neural network (CNN) is a class of deep neural networks that is characterized by shared-weights architecture and translation invariance characteristics, and are therefore often used for image analysis [15].

The effectiveness of learning in CNN models can be improved even further. There are many important factors to consider, such as improving model weight initialization by transfer learning or using data augmentation and dropout as methods of regularization to combat overfitting during model training [16,17,18]. In training CNN models, a large dataset is needed for the model to learn the patterns of features that are complex in detail so that the CNN model can classify those features, achieving an appropriate classification performance [19,20]. Therefore, the researchers often try to reduce time to learn useful features from the dataset by CNN model by fine-tuning the hyperparameters of the adjustment methods mentioned above. This enables learning with a reduced learning time and therefore can support efficient learning from small- and medium-sized datasets [21]. This can efficiently support the learning of small- and medium-sized datasets. In 2018, Rajaraman et al. aimed at developing a CNN model to improve the performance of the computer aided diagnosis (CAD) system to detect malaria cells using deep learning with a malaria dataset, which obtained malaria cell images from the thin blood smears. This research used a deep learning technique to help diagnose malaria-infected and uninfected blood cells. The objective of developing a CAD system intends to help with the screening of malaria patients, thus reducing the workload of practitioners in diagnosing large numbers of patients. It also helps to enhance the accuracy of malaria detection by radiologists with little experience in diagnosing this disease [15]. The model was developed to improve the hyperparameter tuning of the optimizer which were originally a stochastic gradient decent (SGD) and Adam, with adjustment of the learning rate and the use of CNN architecture such as VGG-16, ResNet50, Xception using rectified linear unit (ReLU) [1]. In 2019, the accuracy achieved using Mish activation function was 1.671% more than the accuracy of the model that used ReLU on the dataset CIFAR 100, which is one of the most effective activations compared to the state of the art of activation function performance between (Mish) and (Swish) activations function that were developed in 2018. Mish is still more than 0.494% more effective, validated with a 70-item benchmark dataset [22]. In 2015, the optimizer named Nesterov accelerated adaptive moment estimation (Nadam) was developed from Adam and was combined with the Nesterov accelerated gradient that was developed in 2014, and is used in the development of this research [23].

The performance of Xception [24] is slightly better than that of Inception-v3 [25] on the ImageNet dataset [26]. However, these higher levels of performance do not result from the expanded capacity but are instead due to the more effective use of model parameters, as the number of parameters in the architecture of Xception is the same as that of Inception-v3. In 2018, a research study using VGG-16 model in combination with transfer learning was conducted to automatically classify single cells in thin blood smears on standard microscope slides consisting of uninfected and infected samples, amounting to 27,578 single cell images. Adjustment of the size of the images was applied in the experiment, in which the width and length was 44 × 44 pixels, with three color channels (red, green, blue) from Chittagong Medical College Hospital, Bangladesh, to develop the CAD system to diagnose malaria with an accuracy of 97.37% [15]. In 2017, CNN and support vector machine (SVM) were used to diagnose malaria. In the research, 1034 infected cell images and 1531 uninfected cell images were collected from the University of Alabama at Birmingham. The research divided the malaria dataset into two sets of approximately equal size, by which it was shown that SVM provided accuracy of 91.66%, and CNN provided accuracy of 95% [27]. In 2020, ResNet was used to increase the effectiveness of training on the dataset consisted of 1,182 blood cell images at three different magnifications of 200x, 400x and 1000x with a 750 × 750 pixel resolution collected from microscopic observation. For the creation of the CNN model, the dataset was divided into 80% for training and another 20% for validation, and an accuracy of 98.08% was achieved [28].

Masud et al. aimed at developing a CNN model by fine-tuning the hyperparameter of the pretrained model and improving performance by using cyclical learning rates-triangular2, which finds the best learning rate of SGD to improve the performance for malaria detection [29]. Vijayalakshmi et al. proposed CNN models (VGG16, VGG19) with support vector machines (SVM) to determine the stages of parasite infection and improved the training time by using pre-trained CNN models and the transfer learning technique [30]. The aim was to improve the architecture by using state-of-the-art activation function (Mish) to increase the performance of the CNN model. The optimal effectiveness of the model was proposed to be achieved by using other optimizers, such as, SGD and Nadam [31]. The contribution of the research [32] was aimed at developing a CNN model to fine-tune the hyperparameter of the pre-trained model by using transfer learning.

This paper used the above-mentioned powerful techniques to develop the research. The contribution of the proposed work aims at the improvement of the CNN model and fine-tuning it to develop a CAD system for the detection of malaria by applying Mish, which is considered to be an effective activation function. This research was conducted to examine the use of Xception architecture with a combination of Mish and Nadam. If ReLU is replaced by Mish for use inside Xception, the enhancement of the performance of the image classification may be achieved, particularly when compared with the original Xception architecture, as well as other types of CNN architecture. In sum, the proposed deep learning model utilized Xception in combination with Mish and Nadam and this method achieved an accuracy of 98.86% on the malaria detection task. Hence, it is feasible to employ the presented deep learning model for malaria detection.

## 2. Materials and Methodology

### 2.1. Methodology

**Figure 2:**
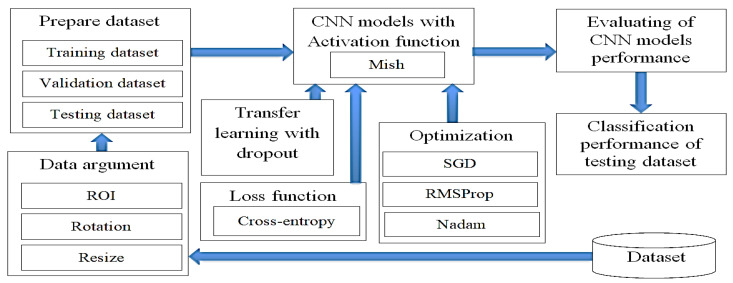
The research methodology.

The methodology can be divided into five parts. The first part used image processing techniques, such as region of interest (ROI) selection [33] that is commonly used in many application areas. It divides images into sections according to the borders of an object under consideration. The target of ROI selection is to change the images so that they are more meaningful and easier to analyze. Normally, ROI is used to find a greater accuracy of the position and boundary of the objects inside the images. The result of ROI is a set of images or contours which are extracted from images, and data augmentation for improving the malaria cell dataset. The second part prepared a malaria cell dataset to improve CNN models and was split into three datasets, containing a training, validation and testing dataset. The third part involved the Mish activation function, dropout techniques and transfer learning to develop the ability of CNN models to detect malaria. The fourth part used the Cross-entropy loss function and three optimizer methods, including SGD [34], RMSProp [35] and Nadam [23], to examine the prediction performance of CNN models in the classification of malaria cells from the malaria cell dataset. The fifth part evaluated the CNN models’ effectiveness for malaria cell classification from the malaria cell dataset, as shown in Figure 2.

### 2.2. Dataset

**Figure 3:**
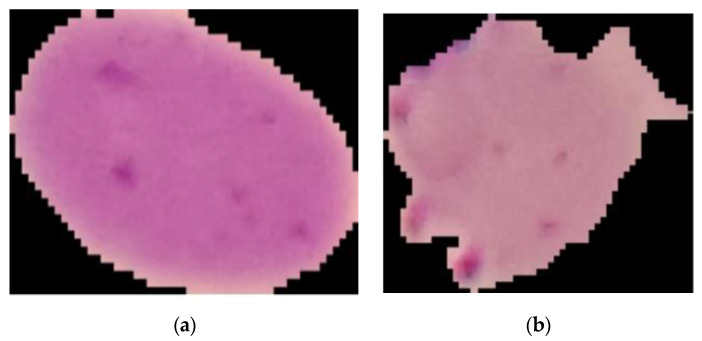
Normal red blood cells (**a**) and red blood cells infected with malaria (**b**).

This research utilized a set of graphic data as a sample to develop an efficient CAD system to enhance the precision and minimize the time required for the identification of malaria, as well as to reduce the medical professional’s burden of screening malaria patients. The data were collected from a thin blood smear on a slide containing malaria from the hospital by using a microscope. The total sample comprised 201 patients, of which 151 were infected and 50 patients were not. This open access dataset contained normal RBCs and RBCs infected with malaria images, which were different in shape and color, and was stored in the database of the National Library of Medicine (NLM). The Lister Hill National Center for Biomedical Communications used this dataset for the development of a CAD system that could be used with an Android system [1,15]. This research selected 7000 images, of which 4500 images were of infected slides and 2500 images were of uninfected slides, as shown in Figure 3.

#### Data Augmentation

After the malaria-infected red blood cell images were tilted 90, 180 and 270 degrees by rotation respectively, Figure 6 was produced. This adjustment involved data enhancement, also known as data augmentation, which came from the existing dataset and produced more data for the network to learn. This method could make up for the lack of data for the training, validation and testing of a CNN model, as this involves a set of altered images different from the original ones. In the case of data enhancement, this could solve the issue of insufficient data and improve the accuracy of convolutional neural network training [36,37].

### 2.3. The Structure of CNN Model

#### 2.3.1. Convolutional Layer

The convolutional layer is employed for processing to detect line images by using the Sobel technique or other techniques. The Sobel mask operation involves various rounds of image convolution with filters in horizontal and vertical directions. Additionally, each image has a pixel density, which is called the resolution or contrast, but will also have some different pixels. Pixels are used to determine image qualification and displays. If images have more pixels, they will have a high resolution. In digital images, computer graphic images, or bitmap images, the network will display a square table of pixels or continuous pixels. Pixels are only one of the smallest points in digital images, such as display papers or other multimedia. Each pixel will have its own space corresponding to its coordinates. The intensity of each pixel is different in a colors image system. Colors are shown as a color intensity, such as red, green, blue, yellow and black. The convolutional layer is flattened, and its function is to transform a multidimensional vector into a one-dimensional vector [38].

#### 2.3.2. Pooling Layer

The pooling layer is one of the concepts used for extracting important features of convolutional neural networks, in order to reduce the dimensions of the data by combining the outputs of neuron clusters at one layer into a single neuron in the next layer, which reduces the repeatability of neural network features and preserves information concerning the key features through this algorithm. It can also increase its network instruction cycle, as well as prevent the problem of overfitting in the analysis of high complexity problems. Common pooling layer calculation methods include the maximum pooling method, average pooling method and the Gaussian pooling method, the latter of which is widely used and represents the largest pooling method. The following provides a detailed description of its differences [39].

#### 2.3.3. Activation Function

ReLU is a non-linear activation function. It is used in multi-layer neural networks or deep neural networks, the output of ReLU is the maximum value between zero and the input value, which effectively removes negative values from an activation map by setting them to zero [40].

#### 2.3.4. Fully Connected Layer

**Figure 4:**
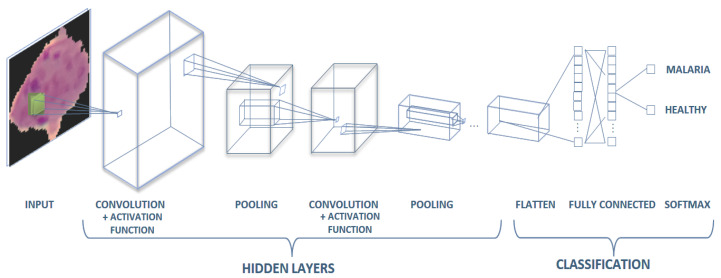
Convolutional neural network (CNN) model.

Fully connected layers in a CNN are those layers in which all the inputs from one layer are connected to every activation unit of the next layer, which takes the end result of the convolution, pooling layer and reaches a classification, feature extraction is performed, and is conducted in the final layer of the neural network; in other words, the fully connected input layer consists of weight values from perceptrons, depending on the structure defined. Fully connected output layer provides the final probabilities for each label [41], as shown in Figure 4.

#### 2.3.5. Softmax

Softmax is often applied to the last layer of the neural network to provide the output as a probability to calculate the Negative Log Likelihood as a cross-entropy loss, such as in multiple class classification. Figure 4 illustrates the full structure of the CNN model [42].

### 2.4. Optimization Methods

#### 2.4.1. Transfer Learning

**Figure 5:**
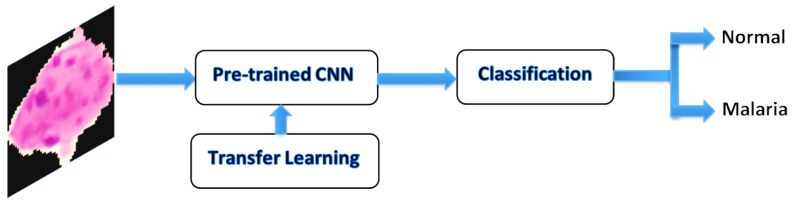
Convolutional neural network (CNN) transfer learning.

Transfer learning is a method whereby a neural network model is first trained on a problem similar to the problem that is being solved, which employs the weight of the model and learned from a large dataset such as an ImageNet dataset. This technique is flexible, allowing the use of pre-trained models directly as feature extraction preprocessing, and integrated into entirely new models. It has also been applied to cancer subtype discovery [1,31]. Figure 5 shows an example of the transfer learning process with the CNN model using pre-trained weights.

#### 2.4.2. Dropout Technique

The dropout technique is very simple. An example structure model is as follows. The model starts with ignoring some random neurons in the CNN model. Therefore, the ignored neurons will not send the data to other neurons. The dropout specification of 0.5 on the fully-connected layer will ignore 50% of neurons in that layer [43]. Ignoring neurons in each layer randomly may seem like a bad idea, but in truth, this technique works well because it does not completely block the input signal, but only some connections between neurons. This random close selection will prevent co-adaptation and is effective in reducing overfitting because it makes the model “easier” [44,45].

#### 2.4.3. Optimizers

Gradient descent is used to minimize some functions by iteratively moving in the direction of steepest descent as defined by the negative of the gradient, which is an optimization algorithm that is used to improve deep learning and neural network-based models by minimizing the cost function. It is susceptible to local minima since every data instance from the dataset is used for determining each weight adjustment in the neural network. Gradient descent is used to update the hyperparameters of optimizers, which is used to control the learning process of neural networks [46].

1. Stochastic gradient descent (SGD) is a very common optimization algorithm in neural network model training. This algorithm is based on the gradient descent algorithm. SGD proposed on this basis only considers one sample at a time, which makes the direction of each iteration not necessarily the direction of the overall optimization of the model [34].
  (1) $$u=u-\eta .\nabla_{u}g(u;x^{i};y^{i})$$
  SGD is an optimization technique, which minimizes a loss function in performing a gradient descent step sample by sample. The learning rate of SGD is 0.001 (*η*), the training using the label is *y*^i^ and the input is ꭓ^i^. The cost function of the calculating gradient is μ.
2. RMSprop is the root mean square prop algorithm, which can speed up the gradient descent like the momentum method. In order to further optimize the problem of excessive swing amplitude in the update of the loss function. Hinton did not publish RMSprop in a formal academic paper, but it still became one of the most popular gradient descent optimization algorithms for deep learning. Normally, this has a score of 0.9 [35]. The formula is shown below.
  (2) $$\theta_{t+1}=\theta_{t}-\frac{\eta }{\sqrt{Ε[g^{2}]t+\varepsilon }}gt$$
  The study used Hinton’s input (γ, or rho) to determine the value (0.9) for the solving fraction at time step t [34]. θ_t+1_ is the decaying average and [*g*^2^]*t* is the diagonal matrix and the learning rate of RMSprop is 0.001 (*η*).
3. Nadam [23] involves robust learning from the previous time point, which has many direct impacts for the gradient descent to update weights. Nadam based on the Adam optimizer [47] is a popular optimizer because it combines the strengths of each optimizer and then removes the weakness points of the Adagrad [48] decaying learning rate, producing the model and enabling studying to continue, and it is also faster than the gradient descent and reduces the discontinuous problems of the parameters.
  (3) $$\theta_{t+1}=\theta_{t}-\frac{\eta }{\sqrt{\overset{}{v_{t}}}+\varepsilon }\left(\beta_{1}{\overset{}{m}}_{t}+\frac{\left(1-\beta_{1}\right)gt}{1-\beta_{1}^{t}}\right)$$
  The learning rate of Nadam is 0.002 (*η*); the objective function (θt) using *ε* = *le^−08^* and *β_1_* = 0.9, based on the work in [34] using *v_t_* and *m_t,_* which represents the improved efficiency at time step *t* of the optimizer (Nadam).

#### 2.4.4. Mish Activation Function

Activation functions that are still widely used include Swish, PReLU, ELU, TanH, Sigmoid and Leaky ReLU. In this paper, a powerful activation function known as Mish [22] is proposed to use with the Xception model, and a conclusion is drawn that the accuracy of Mish is 0.494% higher than Swish and 1.671% higher than ReLU on the malaria detection task. This may be because Mish has the features of self-selecting gates, which are beneficial compared to other activation functions like ReLU (point-to-point functions). Mish can be implemented using any of the CNN frameworks and it guarantees non-monotonic and smooth output for each point, therefore improving the results. The input of Mish is indicated by variable (*k*) in Formulas (4) and (5).

(4) ReLU(k)=max(0,k)

(5) Mish(k)=k*tanh(softplus(k))

#### 2.4.5. Loss Function

The loss function employed for classification involves indicators. It is the only number that shows how well a specific model works by comparing the output of the model. Cross-entropy or the cost function was used to assign the sample to be computed, which comprised of a learning rate (*η)* and (ε) indicates node internal neuron output, where L indicates the output of the loss function. These parameters are independent of each other. The most commonly used correction functions are loss functions, such as mean square error (MSE), mean absolute error (MAE) and Cross-entropy [49]. This experiment used (Cross-entropy) as shown in Formula (6):

(6) $$L=-\frac{1}{n}[\eta ln\varepsilon +(1-\eta )ln(1-\varepsilon )]$$

### 2.5. Model Performance Evaluation

The confusion matrix shows the result of the classification and is divided into two classes. Each value is displayed in each row to show the amount of data contained inside the label’s classes. This research applied these parameters to estimate the CNN model’s performance by using true positive (TP), which means that the predicted results are positive and the actual value is positive, while true negative (TN) means that the predicted result is negative and the actual value is negative. Furthermore, false positive (FP) means that the predictive result is positive, but the actual value is negative, and false negative (FN) means that the predicted result is negative, but the actual value is positive. The Formulas (7)–(11) are based on the work presented in [50,51,52].

1. The accuracy, regardless of whether it is actually a positive sample or a negative sample, calculates the ratio of predicted to actual values. The Formula (7) is shown below.
  (7) $$Accuracy=\frac{TN+TP}{TN+TP+FN+FP}$$
2. The precision is the ratio of all positive samples that are actually positive, as shown in Formula (8).
  (8) $$Precision=\frac{TP}{FP+TP}$$
3. The recall is the ratio of positive predictions among all positive, as shown in Formula (9).
  (9) $$Recall=\frac{TP}{FN+TP}$$
4. The F1 measure is a metric employed to describe the classification performance of the system. It is calculated through the recall and precision rate, as shown in Formula (10).
  (10) $$F1measure=\frac{2TP}{2TP+FP+FN}$$
5. The effectiveness of CNN model testing is the true prediction divided by the amount of the testing dataset, the result employed to describe the classification performance of the CNN model, as shown in Formula (11) and the results shown in Table 11.
  (11) $$The\;effectiveness\;of\;CNN\;model\;testing=\frac{True\;prediction\;numbers}{Images\;numbers}\times 100\%$$

## 3. Implementation Details

This study involved the development of a CAD system for detecting malaria in thin blood smear images with deep learning techniques. Below we provide the description of the implementation environment that included software and hardware. The details are shown in Table 1.

This research uses six CNN models that are popular with computer vision in image classification, including AlexNet [53], VGG-16 [54], NasNetMobile [55], ResNet-50 [56], Inception-V3 [25] and Xception [24], which allow for more efficient optimization of parameters, including the optimizer, batch size, learning rate, activation function, dropout, loss function, etc. In this experiment, the optimizer uses SGD with a learning rate of 0.002, RMSProp with a learning rate of 0.001 and Nadam with a learning rate of 0.002. These values are based on the research presented in [34,57,58].

For dropout, 0.5 and 20 batch sizes are specified, which are used to increase training speed. In addition, the activation function includes ReLU and Mish, which is one of the most effective state-of-the-art approaches, the loss function is cross-entropy, and the Softmax function takes the weights and converts them into the probability to predict malaria [62,63,64]. The iterations are 50 epochs and the output layer of the CNN model in this research has two classes, which consist of an infected status and uninfected status, as shown in Table 2.

In addition to the gradient derived from the cost function, there is another parameter that we need to optimize when training the gradient descent algorithm: the learning rate, or alpha, for an optimization algorithm. Choosing a learning rate directly affects the performance of the gradient descent algorithm.

### 3.1. Dataset Setting

**Figure 6:**
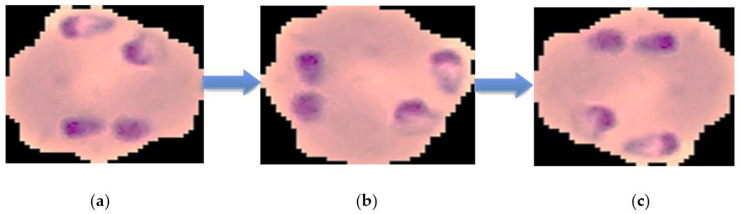
The results of malaria images in the dataset using a region of interest (ROI) boundary and the rotation technique: (**a**) the malaria-infected red blood cells were tilted 90 degrees by rotation; (**b**) the malaria-infected red blood cells were tilted 180 degrees by rotation; and (**c**) the malaria-infected red blood cells were tilted 270 degrees by rotation.

Due to the thin blood smear film, it is not appropriate for training CNN models, therefore it is required to adjust the thin blood smear film images. Techniques to increase the number of images in a dataset through a rotation technique are popular and are used to increase the effectiveness of small data sets, but typically rotate by no more than 90 degrees. In this study, the image is assigned an angle of 0 to 270 degrees randomly using the shuffle sampling technique together with the rotation in the development. These methods increased the malaria image dataset to 7000 images, consisting of original images from the thin blood smear, and images obtained by the rotation and sampling techniques to reduce data duplication [65,66]. The data enhancement flip diagram is shown in Figure 6. The image was constructed such that the data had a normalization value between 0 to 1, by changing the range of pixel intensity values. In this research, we resize the images to suit the CNN model’s structure used in the development CAD by adjusting the matrix size to 224 × 224 × 3 and 299 × 299 × 3 with blue, red and green colors (or an RGB color system). The malaria dataset was split into training 80%, validation 20% and the final model was applied to 700 images (or 10% of the total number images) to test the CNN model. The research used ROI to detect the image boundaries, which does not affect other parts of the image [67,68].

### 3.2. Xception Architecture, Activation Function (Mish) and Revision of the Model

The continuous improvement of CNN architecture enables more accurate image recognition. The Xception architecture was built upon a variety of essential principles, including a convolutional layer, a depth-wise convolutional layer, and a separable convolutional layer. Furthermore, the activation function is required for this architecture, wherein Mish is an innovative activation function, which provides an alternative to commonly used activation functions such as ReLU. This subsection introduces the updated Xception architecture, including the latest Xception with Mish design [22,24].

#### 3.2.1. Xception Architecture

**Figure 7:**
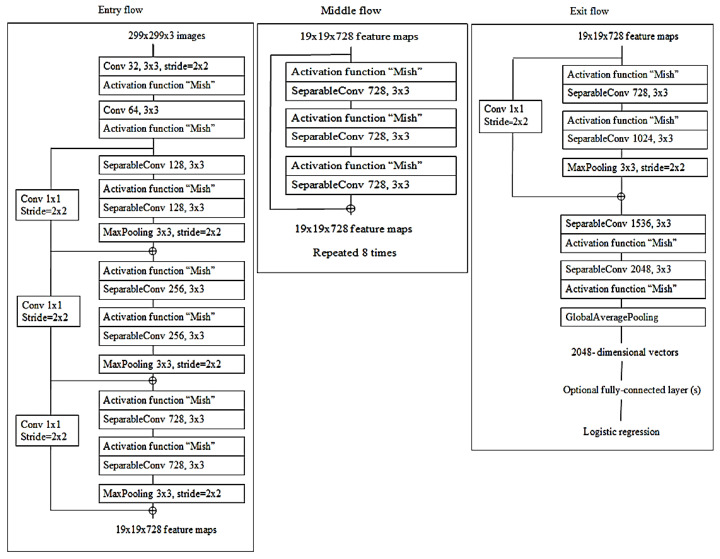
The architecture of Xception.

Xception is a concept founded on the original Inception design that generates cross-channel and spatial relationship similarities within CNN’s feature maps that can be fully detached. The framework uses cross-channel correlations by splitting input data in four ways to obtain a 1 × 1 convolution size and conducts average pooling, and then maps 3 × 3 convolution size correlations and forwards them for concatenation [24], as shown in Figure 7.

**Figure 8:**
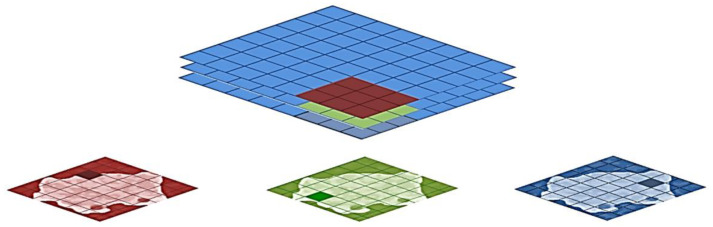
Xception used the depth-wise convolution to split the image by using a 3 × 3 filter linked to 1 × 1 point-wise convolutions to build a linear convolution.

The depth-wise separable convolution proposed was also able to identify eye-catching objects in image detection by using 3 × 3 convolution kernel size. Point-wise convolution, commonly known as 1 × 1 convolution and abbreviated as PW, is mainly used for data dimensionality reduction and parameter reduction. In Xception, PW is used to change three feature maps into six feature maps, which enriches the features of the input data [69], as shown in Figure 8.

#### 3.2.2. Convolution Kernel Replacement

Even with PW, due to the 3 × 3 and 1 × 1 convolution kernel parameters, directly calculating such a large amount is still very difficult, the training time is quite long and Xception has to conduct re-optimization by replacing multiple large convolution kernels with multiple small convolution kernels [54].

(12) $$F_{m}=MaxPooling\left(F_{i},v\right)$$

where *v* represents the max-pooling filter. The output attribute map describes *F_m_*, which is sorted by shape and size, where every *F_m_* saves the highest value of Fi in the input attribute map [70]. Each module is positioned equally in relation to the original Xception with Mish architecture, as demonstrated in Figure 8. At the activation function point, only ReLU is substituted with a Mish. An additional Mish is appended after global average-pooling and prior to logistic regression as a small change. For the grouping of images, the original Xception model is ideal. Still, sustained development must involve classification operation enhancement.

**Figure 9:**
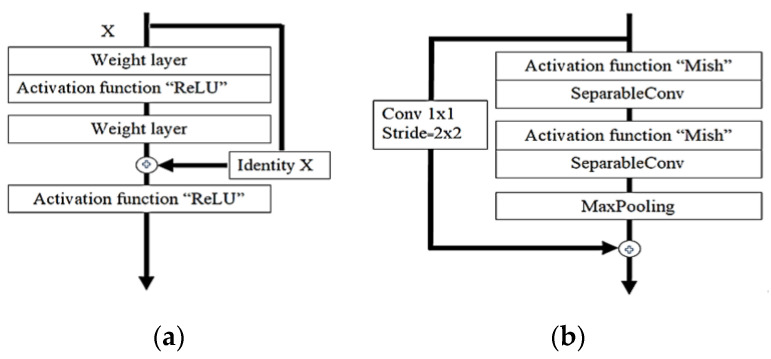
Residual connection diagram procedure showing (**a**) the residual connections of ResNet architecture; (**b**) Xception used residual connection to avoid the problem of vanishing gradients.

To evaluate the performance, we examined the Mish activation function. Accordingly, the design for the original Xception is used as the basis for the novel model, though it employs the Mish activation function to boost the performance of image classification.

(13) $$Ov=f\left(Iv,\left\{P_{i}\right\}\right)+Iv$$

where *Iv* represents the input channels and *Ov* represents the output channels for the layers. The estimate of f (*Iv,{P_i_*}) notifies the outstanding mapping to be understood. The capacity to avert signal mitigation through the conversion of many stacked nonlinearities is one advantage of the residual link [71], as shown in Figure 9.

## 4. Experimental Results

Table 3, Table 4, Table 5, Table 6, Table 7 and Table 8 show the CNN model’s performance using Mish and three optimizers. Table 3 illustrates the malaria disease detection effectiveness of traditional NasNetMobile, with ReLU compared to NasNetMobile, which uses Mish. The optimal results of NasNetMobile were achieved by the use of Mish and Nadam, with the F1 measure rate at 90.99%, the recall rate was 90.98%, the precision rate was 91.01% and the accuracy rate was 91%, which had an execution time usage of 72 min 12 s. NasNetMobile combined with ReLU and SGD offered the lowest effectiveness; an F1 measure rate of 78.64%, a recall rate of 78.63%, a precision rate of 78.64% and an accuracy rate of 78.64% were obtained.

For Inception-V3, the optimal results were achieved by using Mish with Nadam, and the F1 measure rate was 95.20%, the recall rate was 95.21%, the precision rate was 95.21% and the accuracy rate was 95.21%, which had an execution time usage of 67 min 12 s. In addition, for Inception-V3 combined with ReLU and SGD offered the lowest effectiveness, an F1 measure rate of 87.28%, a recall rate of 87.31%, a precision rate of 87.28% and an accuracy rate of 87.29% were obtained. Table 4 demonstrates the results of these models.

Table 5 illustrates the optimal effectiveness of Xception for the detection of malaria using Mish and the optimizer method, which can improve the performance of Xception. In addition, this research used Mish and Nadam employing Xception to predict malaria with an F1 measure rate of 99.28%, a recall rate of 99.28%, a precision rate of 99.29%, and an accuracy rate of 99.28%, which had an execution time usage of 125 min 29 s.

**Figure 10:**
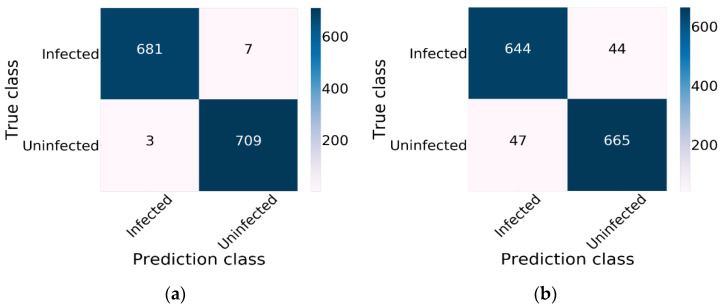
The result of Xception obtained by using a confusion matrix: (**a**) The results of Xception using Nadam and Mish; (**b**) the results of Xception.

Xception combined with ReLU and SGD provided the lowest effectiveness. An F1 measure rate of 93.49%, a recall rate of 93.50%, a precision rate of 93.49% and an accuracy rate of 93.50% were obtained. The performance of Xception using Nadam and Mish is demonstrated in Figure 10, which displays the effectiveness of CNN model training using a training dataset. The confusion matrix result for Xception using Nadam and Mish is demonstrated in Figure 10a. Xception predicted an uninfected status for 709 images and an infected status for 681 images of an infected status and did not correctly predict malaria for 10 images. Figure 10b demonstrates the results of Inception-V3; this model correctly predicted an uninfected status for 665 images and an infected status for 644 images.

Table 6 illustrates the malaria disease detection effectiveness of traditional AlexNet, with ReLU compared to AlexNet, which uses Mish. The optimal results of AlexNet were achieved by the use of Mish and Nadam where the F1 measure rate was 82.70%; the recall rate was 82.78%; the precision rate was 82.92%; and the accuracy rate was 82.71% and had an execution time usage of 15 min 15 s. AlexNet combined with ReLU and SGD provided the lowest effectiveness: an F1 measure rate of 76.05%, a recall rate of 76.05%, a precision rate of 76.07% and an accuracy rate of 76.07% were obtained.

Table 7 illustrates the malaria disease detection effectiveness of traditional VGG-16, with ReLU compared to VGG-16, which uses Mish. The optimal results of VGG-16 were achieved by the use of Mish and Nadam, where the F1 measure rate was 84.99%, the recall rate was 85%, the precision rate was 84.99% and the accuracy rate was 85%, which had an execution time usage of 51 min 12 s. For VGG-16 combined with ReLU and SGD the lowest effectiveness was provided: an F1 measure rate of 78.83%, a recall rate of 78.83%, a precision rate of 78.86% and an accuracy rate of 78.85% were obtained.

Table 8 illustrates the malaria disease detection effectiveness of traditional ResNet-50, with ReLU compared to ResNet-50, which uses Mish. The optimal results of ResNet-50 were achieved by the use of Mish and Nadam, where the F1 measure rate was 93.07%, the recall rate was 93.10%, the precision rate was 93.13% and the accuracy rate was 93.07%, and which had an execution time usage of 49 min 52 s. ResNet-50 combined with ReLU and SGD provided the lowest effectiveness: an F1 measure rate of 86.70%, a recall rate of 86.78%, a precision rate of 86.96% and an accuracy rate of 86.71% were obtained.

## 5. Discussion

To improve CNN model performance, we can use various optimizers, activation functions and image processing techniques to extend the original malaria dataset. Furthermore, the image classification ability can be boosted by data augmentation approaches. The parameters utilized to adjust the function of each optimizer are employed in approaches. The arguments of Nadam comprised of the learning rate, epsilon, beta_1, and beta_2. The arguments of RMSprop comprised of the learning rate, momentum, epsilon, rho; the arguments of SGD comprised of learning rate, momentum and Nesterov [54,63], are shown in Table 9.

Xception is defined as a hypothesis based on the Inception, which performs correlations of cross-channels and spatial relations within feature maps of the CNN model. As revealed in Figure 9, devolving more appreciably from the established convolution method with the depth-wise convolution aligned with the point-wise convolution and producing a 1 × 1 convolution kernel size that executes the depth-wise separable convolution enables this. Based on this, Xception was born, and the author called it Extreme Inception.

This experiment faced several limitations. First, the recommended conditions were not possible with the low computer hardware features, indicating the unsuitability of the application software in this assay. Contemporary computer hardware might be feasible for extensive image assessment as it performs to a high degree. The operation of the classification models using several optimization approaches is compared in Table 10. Xception linked with Nadam and Mish was the most accurate of the CNN models, offering an accuracy of 99.28%. Inception-V3 with Nadam and Mish, with a 95.21% accuracy, provided the second-best accuracy. ResNet-50 merged with Nadam and Mish offered the third-highest accuracy of 93.07%. NasNetMobile with Nadam and Mish, with a 91% accuracy. The minimum time was derived from AlexNet combined with ReLU and SGD, which had an execution time usage of 14 min 24 s when comparing the classification models’ time consumption. The next-shortest duration came from ResNet-50 combined with ReLU and SGD, providing an execution time consumption of 48 min 34 s. VGG-16 combined with ReLU and SGD had the third-lowest time consumption of 49 min 51 s. Inception-V3 combined with ReLU and SGD had the fourth-lowest time consumption of 64 min 17 s. NasNetMobile combined with ReLU and SGD had the fifth-lowest time consumption of 63 min 31 s. Xception combined with ReLU and SGD had the sixth-lowest time consumption of 121 min 15 s.

**Figure 11:**
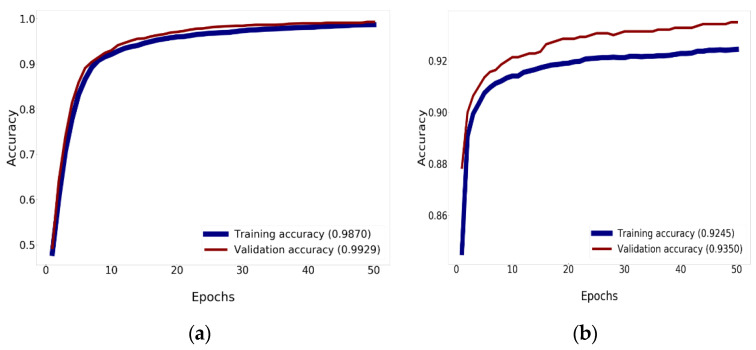
The accuracy history of validation and training: (**a**) Accuracy of training and validation of Xception using Nadam and Mish; (**b**) the accuracy of training and validation of Xception.

**Figure 12:**
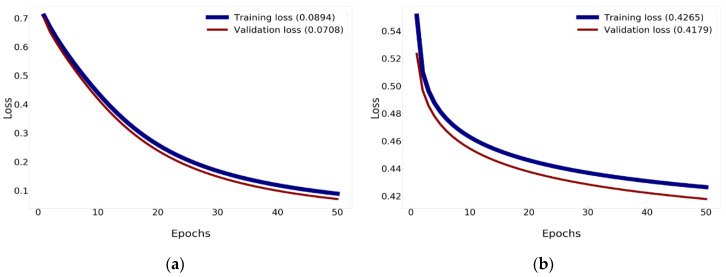
The loss history of validation and training: (**a**) The loss of training and validation of Xception using Nadam and Mish; (**b**) the loss of training and validation of Xception.

To optimize network training, this study specified the parameters of the batch size according to the criteria used for every model of CNN. The amount of samples defined for the training session is the batch size. A higher lot size improves the discovery level of the model. The lot size impacts the usage of GPU memory. When the accessible GPU capacity is not substantial, it is safer to use a lower value. For this study, the accuracy of Mish was higher than the accuracy of ReLU. Mish guarantees the cohesiveness of every point. Mish possesses a lower limit, but there is no higher limit. In fact, the seamless and non-monotronic features also have an influence on the productivity. Analysis of the validation accuracy is shown in Figure 11 and Figure 12.

Figure 11 reveals comparison results for the training and validation accuracy between Xception using Nadam with Mish and the traditional Xception, which uses ReLU, elevating the accuracy to 92.45% for training and 93.50% for validation. Figure 11a shows that Xception can enhance the precision to 98.70% for training and 99.29% for validation. For the training and validation history, 50 epochs are needed, as determined by this research.

The findings of the correlation for validation and training losses between Xception using Nadam with Mish and Xception show a reduction in loss to 0.0894% for training and 0.0708% for validation, as revealed in Figure 12. Xception minimizes the loss to 0.4265% for training and 0.4179% for validation.

**Figure 13:**
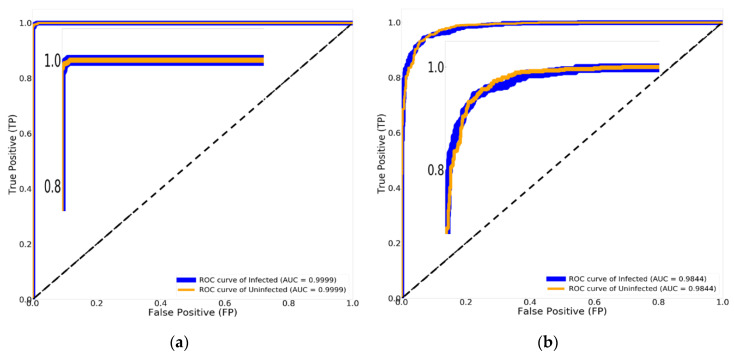
The results of Xception obtained by using the receiver operating characteristics (ROC) curve and area under the curve (AUC): (**a**) Xception combined with Nadam and Mish; (**b**) Xception (traditional method).

Figure 13b shows the AUC of 98.44% with Xception (Traditional method) and the AUC of 99.99% with Xception paired with Nadam and Mish, as shown in Figure 13a. Adjusting the hyper parameters using three optimizing procedures and Mish, while governing the correct values for each optimizing parameter in order to achieve the optimum results, enables the research to affect the stability of traditional CNN models.

Table 11 displays the effectiveness of the CNN model testing with testing dataset, including 315 images of an uninfected status and 385 images of an infected status from the malaria dataset. Erroneous estimates for 3.49% of the uninfected status, or 11 images and 2.85% of the infected status, or 11 images, were produced by the traditional Xception approach; 96.51% of the uninfected status, or 304 images and 97.15% of the infected status, or 364 images, were valid forecasts. Erroneous forecasts of 1.26% for the uninfected status, or four images and 1.03% for the infected status, or four images, were established by Xception combined with Nadam and Mish; real estimates for 98.74% of the uninfected status, or 311 images, and 98.97% of the infected status, or 381 images.

## 6. Conclusions

This study aimed to apply a deep learning model for the detection of malaria. The proposed approach employed Xception, and comparisons were drawn with alternative network models, including Inception-V3 ResNet-50, NasNetMobile, VGG-16 and AlexNet. Malaria causes large numbers of fatalities every year, and poses a particular threat to younger people. The CNN deep learning approach offers a means of producing effective image classification models which might be well-suited to medical applications, such as malaria detection and diagnosis. However, the CNN approach has not yet undergone trials using malaria images, which might support doctors during initial screenings, thereby leading to faster diagnoses, which is the purpose of the research. The classification accuracy of CNN can be improved by the application of an activation function, known as Mish. If Mish is used inside Xception in the place of ReLU, the image classification performance may be enhanced, especially in comparison to the initial Xception architecture, along with other CNN architectures. This paper sought to use a novel Xception modification along with the Mish activation function and Nadam to explore the potential for developing a new screening system which might detect malaria. This system could be trained using benchmark malaria datasets and by applying a technique for augmentation which can improve the quality of the image dataset.

The research methodology consisted of five sections. The first and the second steps required data method preparation, involving data augmentation methods and then split the malaria dataset into three datasets for training, validation and testing. The effectiveness of the CNN model could be significantly enhanced, depending on the number of images involved and the choice of data preprocessing methods used. Some CNN structures are appropriate to use as the dataset training parameters, in order to boost the accuracy and lower the amount of time required. The third step consisted of transfer learning, along with dropout techniques, which were used to make the CNN model more efficient. Dropout served to address the problem of overfitting, while transfer learning helped to enhance the time consumption effectiveness and to achieve a more accurate classification of the images. The fourth step employed the Mish activation function, which can be combined with a loss function based on the concept of cross-entropy, and a number of other optimizer methods, such as SGD, Nadam and RMSprop, in order to establish which CNN model would generate the best prediction performance. The fifth step used a confusion matrix and ROC to evaluate the CNN models’ effectiveness for malaria cell classification.

Training of the model can be conducted using optimization and will depend upon the activation function, the size of the batch and the optimizer. The three optimizer techniques are able to determine whether it is necessary to alter the CNN model learning rate. Studies investigating the activation functions are still being conducted, and in the field of deep learning. Currently, ReLU function is a popular activation function. This situation may be changed, however, by the arrival of Mish. The scale is determined by the activation function for output variable values derived from input variables, while ensuring smoothness at every point. Mish is able to accept one individual scalar for the purpose of making parameter alterations within the network, with no need to enter any scalar. Mish is partly based on the self-gating capacity of Mish, under which the gate is provided with the scalar input. Self-gating makes it possible to replace functions such as ReLU while the parameters of the network remains unchanged. There is no upper bound for Mish, yet a lower bound does exist. Moreover, the smooth and non-monotonic qualities it offers are able to provide enhanced results. A weighting system places emphasis upon those inputs which serve to establish the weighting along with the associated neuron prior to the transfer of this weighting, which will be employed as the input required for the activation function. As the model undergoes training, the original weightings may see changes, as the overall accuracy is gradually improved. This study has certain limitations, for instance, the computer used in the study has inadequate levels of performance when compared to the stated requirements, and therefore it was not possible to employ the application software during the research. Furthermore, the performance of today’s computer hardware is excellent and makes large-scale image analysis feasible.

A summary of the model testing performance is provided in Table 11, where the detection of malaria was accompanied by a 96.85% accuracy when the model applied was the Xception model. In the case of the model which used Xception in combination with Mish and Nadam, the images achieved an accuracy of 98.86%. This model therefore offers the best malaria detection performance, and was shown to be superior to the Xception model. The results in this study enhanced the optimization of CNN models for each of the parameters used in optimization, including the activation function and learning rate and therefore generated a more efficient performance in the CNN model for malaria prediction.

## Figures and Tables

**Table 1:** The implementation environments used to develop a computer-aided diagnosis system.

**System Software and Application**
Operating System: Windows 10 Professional 64 bit ^1^; Cuda-10.2 ^2^, Cudnn-10.2 ^2^, Tensorflow-GPU-1.13.1 [59]; OpenCV-Python-4.2, Scikit-learn 0.21, Theano-1.0.4, H5py-2.9, Pillow-6.1, Python 3.7 and Matplotlib-3.1 [60,61]
**Computer Hardware**
Graphics Processing Unit (GPU): NVIDIA^®^ GTX 1080 TI, 11 gigabytes ^2^; Central Processing Unit (CPU): Intel^®^ I7-6700, 3.40 gigahertz ^3^; Random Access Memory (DDR4): 32 gigabytes; Solid State Drive: 250 gigabytes

^1^ Windows 10 is product name of Microsoft Corporation, Washington, DC, USA; ^2^ NVIDIA^®^ GTX 1080 TI is product name of NVIDIA Corporation, California, CA, USA; ^3^ Central Processing Unit Intel^®^ I7-6700 is product name of Intel Corporation, California, CA, USA.

**Table 2:** The experimental setting of the CNN models for the training and validation process.

CNN	Input Size	Activation Function	Optimizer	Batch Size/Epoch/Dropout	Learning rate of Nadam/RMSProp/SGD
Xception	299 × 299 × 3	Mish, ReLU	Nadam, RMSProp, SGD	20/50/0.5	0.002/0.001/0.002
Inception-V3	299 × 299 × 3	Mish, ReLU	Nadam, RMSProp, SGD	20/50/0.5	0.002/0.001/0.002
ResNet-50	224 × 224 × 3	Mish, ReLU	Nadam, RMSProp, SGD	20/50/0.5	0.002/0.001/0.002
NasNetMobile	224 × 224 × 3	Mish, ReLU	Nadam, RMSProp, SGD	20/50/0.5	0.002/0.001/0.002
VGG-16	224 × 224 × 3	Mish, ReLU	Nadam, RMSProp, SGD	20/50/0.5	0.002/0.001/0.002
AlexNet	224 × 224 × 3	Mish, ReLU	Nadam, RMSProp, SGD	20/50/0.5	0.002/0.001/0.002

Stochastic gradient descent (SGD).

**Table 3:** The effectiveness of NasNetMobile with ReLU and Mish combined with other optimizer.

Activation Function and Optimizer Methods	Accuracy %	Precision %	Recall %	F1 Measure %	Training Time
Nadam and ReLU	88.64	88.65	88.62	88.63	71 min 36 s
Nadam and Mish	91	91.01	90.98	90.99	72 min 12 s
RMSprop and ReLU	83.79	8379	8378	8379	70 min 48 s
RMSprop and Mish	86.79	86.79	86.79	86.78	71 min 22 s
SGD and ReLU	78.64	78.64	78.63	78.64	69 min 31 s
SGD and Mish	80.50	8050	8050	80.49	70 min 27 s

**Table 4:** The performance of Inception-V3 with ReLU and Mish combined with other optimizers.

Activation Function and Optimizer Methods	Accuracy %	Precision %	Recall %	F1 Measure %	Training Time
Nadam and ReLU	93.50	93.50	93.50	93.49	66 min 38 s
Nadam and Mish	95.21	95.21	95.21	95.20	67 min 12 s
Rmsprop and ReLU	90.21	9021	9021	90.20	65 min 30 s
Rmsprop and Mish	92.86	92.86	92.86	92.85	66 min 23 s
SGD and ReLU	87.29	87.28	87.31	87.28	64 min 17 s
SGD and Mish	89.14	89.14	8914	89.13	65 min 03 s

**Table 5:** The performance of Xception with ReLU and Mish combined with other optimizers.

Activation Function and Optimizer Methods	Accuracy %	Precision %	Recall %	F1 Measure %	Training Time
Nadam and ReLU	98.07	98.08	98.05	98.07	124 min 45 s
Nadam and Mish	99.28	99.29	99.28	99.28	125 min 29 s
RMSprop and ReLU	96.50	96.50	96.49	96.49	122 min 43 s
RMSprop and Mish	97.07	97.07	97.06	97.07	123 min 53 s
SGD and ReLU	93.50	93.49	93.50	93.49	121 min 15 s
SGD and Mish	95	95	94.99	94.99	122 min 03 s

**Table 6:** The effectiveness of AlexNet with ReLU and Mish combined with other optimizers.

Activation Function and Optimizer Methods	Accuracy %	Precision %	Recall %	F1 Measure %	Training Time
Nadam and ReLU	81.64	81.85	81.71	81.63	15 min 04 s
Nadam and Mish	82.71	82.92	82.78	82.70	15 min 15 s
RMSprop and ReLU	79.71	79.88	79.77	79.70	14 min 44 s
RMSprop and Mish	80.92	81.14	80.99	80.91	14 min 51 s
SGD and ReLU	76.07	76.07	76.05	76.05	14 min 24 s
SGD and Mish	77.42	77.43	77.43	77.42	14 min 28 s

**Table 7:** The effectiveness of VGG-16 with ReLU and Mish combined with other optimizers.

Activation Function and Optimizer Methods	Accuracy %	Precision %	Recall %	F1 Measure %	Training Time
Nadam and ReLU	84.35	84.35	84.34	84.35	50 min 56 s
Nadam and Mish	85	84.99	85	84.99	51 min 12 s
RMSprop and ReLU	81.64	81.65	81.61	81.62	50 min 28 s
RMSprop and Mish	82.78	82.78	82.77	82.77	50 min 42 s
SGD and ReLU	78.85	78.86	78.83	78.83	49 min 51 s
SGD and Mish	79.71	79.71	79.69	79.70	50 min 17 s

**Table 8:** The effectiveness of ResNet-50 with ReLU and Mish combined with other optimizers.

Activation Function and Optimizer Methods	Accuracy %	Precision %	Recall %	F1 Measure %	Training Time
Nadam and ReLU	92.50	92.59	92.54	92.49	49 min 36 s
Nadam and Mish	93.07	93.13	93.10	93.07	49 min 52 s
RMSprop and ReLU	89.71	89.90	89.77	89.70	48 min 58 s
RMSprop and Mish	91.21	91.36	91.26	91.21	49 min 12 s
SGD and ReLU	86.71	86.96	86.78	86.70	48 min 34 s
SGD and Mish	88.14	88.33	88.20	88.13	48 min 47 s

**Table 9:** Parameters of each optimizer method.

Optimizer	Parameters
Nadam	Learning rate, Epsilon, Beta_1, and Beta_2
RMSProp	Learning rate, Momentum, Epsilon, Rho, Centered
SGD	Learning rate, Momentum, and Nesterov

**Table 10:** Comparison of the effectiveness and time consumption for classification models using Nadam and Mish.

CNN model, Activation Function and Optimizer Methods	Accuracy %	Precision %	Recall %	F1 Measure %	Training Time
Xception	99.28	99.29	99.28	99.28	125 min 29 s
Inception-V3	95.21	95.21	95.21	95.20	67 min 12 s
ResNet-50	93.07	93.13	93.10	93.07	49 min 52 s
NasNetMobile	91	91.01	90.98	90.99	72 min 12 s
VGG-16	85	84.99	85	84.99	51 min 12 s
AlexNet	82.71	82.92	82.78	82.70	15 min 15 s

**Table 11:** The effectiveness of CNN model testing by using Xception with Nadam and Mish.

Models	Optimizers	Classes	Images Numbers	True Prediction Numbers	False Prediction Numbers	Effectiveness %
Xception (Traditional method)	SGD	Uninfected	315	304	11	96.51%
		Infected	385	364	11	97.15%
		Overall	700	564	22	96.85%
Xception with Mish	Nadam	Uninfected	315	311	4	98.74%
		Infected	385	381	4	98.97%
		Overall	700	692	8	98.86%
